# Supplementary material for: A cross-environment comparison of nontuberculous mycobacterial diversity
Source: Appl Environ Microbiol. 2025 Dec 8;92(1):e01552-25. doi: 10.1128/aem.01552-25 (PMC12838349; doi:10.1128/aem.01552-25)
Supplement: Figure S1 — Map of soil and premise plumbing biofilm collection sites. [file aem.01552-25-s0001.docx]

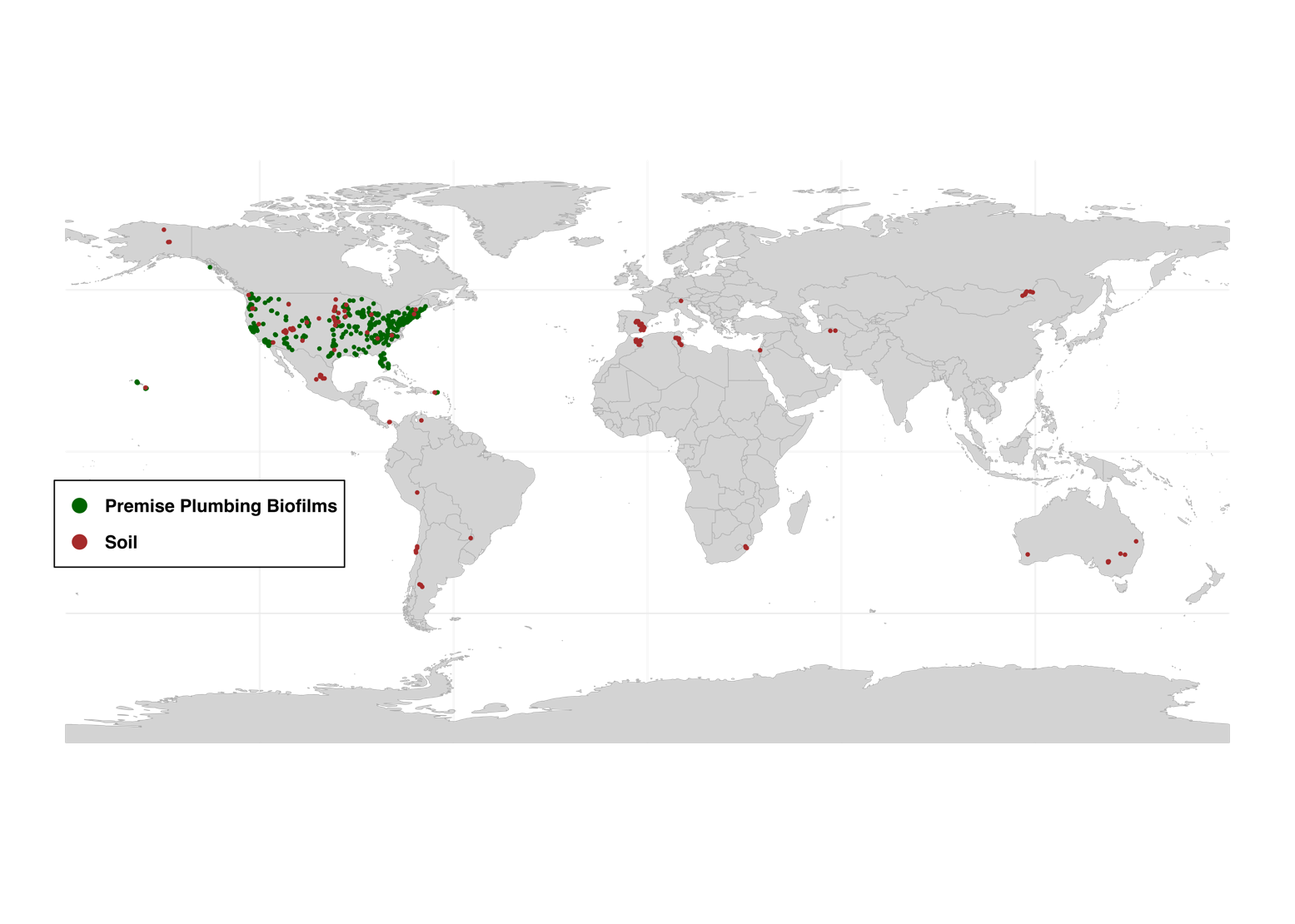


Supplementary Figure 1: World map showing the geographic distribution of premise plumbing biofilm (N= 637), and soil (N=143) sampling sites included in this study, shown in green and brown, respectively. Additional information on sampling locations can be found in Gebert et al (2018) and Walsh et al (2019).
